# Degradation-driven changes in fine root carbon stocks, productivity, mortality, and decomposition rates in a palm swamp peat forest of the Peruvian Amazon

**DOI:** 10.1186/s13021-021-00197-0

**Published:** 2021-10-29

**Authors:** Nelda Dezzeo, Julio Grandez-Rios, Christopher Martius, Kristell Hergoualc’h

**Affiliations:** 1 Center for International Forestry Research (CIFOR), c/o Centro Internacional de la Papa (CIP), Av. La Molina 1895, La Molina, Apdo Postal 1558, 15024 Lima, Peru; 2 Venezuelan Institute for Scientific Research (IVIC), Caracas, Venezuela; 3 Universidad Nacional de la Amazonia Peruana (UNAP), Loreto, Peru; 4 Center for International Forestry Research (CIFOR), Bonn, Germany

**Keywords:** Belowground biomass, Decomposition, Necromass, Peatland, Peru, Tropical regions

## Abstract

**Background:**

Amazon palm swamp peatlands are major carbon (C) sinks and reservoirs. In Peru, this ecosystem is widely threatened owing to the recurrent practice of cutting *Mauritia flexuosa* palms for fruit harvesting. Such degradation could significantly damage peat deposits by altering C fluxes through fine root productivity, mortality, and decomposition rates which contribute to and regulate peat accumulation. Along a same peat formation, we studied an undegraded site (Intact), a moderately degraded site (mDeg) and a heavily degraded site (hDeg) over 11 months. Fine root C stocks and fluxes were monthly sampled by sequential coring. Concomitantly, fine root decomposition was investigated using litter bags. In the experimental design, fine root stocks and dynamics were assessed separately according to vegetation type (*M. flexuosa* palm and other tree species) and *M. flexuosa* age class. Furthermore, results obtained from individual palms and trees were site-scaled by using forest composition and structure.

**Results:**

At the scale of individuals, fine root C biomass in *M. flexuosa* adults was higher at the mDeg site than at the Intact and hDeg sites, while in trees it was lowest at the hDeg site. Site-scale fine root biomass (Mg C ha^−1^) was higher at the mDeg site (0.58 ± 0.05) than at the Intact (0.48 ± 0.05) and hDeg sites (0.32 ± 0.03). Site-scale annual fine root mortality rate was not significantly different between sites (3.4 ± 1.3, 2.0 ± 0.8, 1.5 ± 0.7 Mg C ha^−1^ yr^−1^ at the Intact, mDeg, and hDeg sites) while productivity (same unit) was lower at the hDeg site (1.5 ± 0.8) than at the Intact site (3.7 ± 1.2), the mDeg site being intermediate (2.3 ± 0.9). Decomposition was slow with 63.5−74.4% of mass remaining after 300 days and it was similar among sites and vegetation types.

**Conclusions:**

The significant lower fine root C stock and annual productivity rate at the hDeg site than at the Intact site suggests a potential for strong degradation to disrupt peat accretion. These results stress the need for a sustainable management of these forests to maintain their C sink function.

**Supplementary Information:**

The online version contains supplementary material available at 10.1186/s13021-021-00197-0.

## Background

The Pastaza-Marañon foreland basin in the Peruvian Amazon harbors one of the largest areas of peatlands in the tropics [1]. Its peatlands are important carbon (C) sinks [2, 3] and store an estimated 3.14 Pg C in soil and aboveground biomass [4] which is nearly 50% of the C stored in aboveground biomass for the entire country (6.9 Pg C) [5]. They also host a unique biodiversity [6] and play a significant role in regulating hydrological cycles [7]. In the region, peat-forming vegetation communities are for the most part (78%) swamp forests (permanently or semi-permanently flooded forests) dominated by the palm *Mauritia flexuosa*, a dioecious species that can establish nearly monodominant stands [8–10]. In these swamp forests, *M. flexuosa* is the most important species according to the importance value index (IVI) which considers species relative abundance, dominance and frequency [10]. Nevertheless, trees are also significant in this ecosystem, with some species such as *Tabebuia insignis* ranking second or third in IVI [10]. *M. flexuosa* in addition to sequestering C, is considered a keystone species which plays an essential role in various ecological dynamics as it provides a diversity of resources (e.g., food, habitat) for numerous species [11]. Its fruit is highly demanded locally for commercial and subsistence purposes but is often and extensively collected by felling adult *M. flexuosa* females [12, 13]. Local communities cut the palms because their height (often up to 40 m) and the slippery nature of their bark render their climbing difficult [14]. This unsustainable harvesting practice not only undermines the economic potential of *M. flexuosa* for rural communities [15], it also induces a male dominance in degraded stands [13, 16], modifies the structure and floristic composition of the forest [10, 12, 17] and likely disrupts a wide range of ecological patterns and processes, including belowground C dynamics [18, 19].

In the tropics, peatland ecosystems store approximately 90% of their C belowground [1, 4, 10]. The sustained accumulation of C in the soil results from the imbalance between high plant productivity rates and slow decay rates of the organic matter in waterlogged conditions [20–24]. In tropical forested peatlands of Southeast Asia, fine roots (i.e., roots smaller than 2 mm in diameter) account for a smaller portion of plant productivity (17%) than leaves (53%) and wood (30%) [25] while the contribution of coarse roots is considered minimal [26]. On the other hand, the slow decomposition rate of fine roots renders them as one of the main components forming the peat together with woody material [26, 27]. Fine roots which form the most active part of the root system also play important roles in other ecosystem C dynamics and plant functioning by e.g., supporting plant growth and facilitating water and nutrient acquisition [28].

Tree species in wetlands adapt their root system to cope with anoxic conditions, by modifying their morphology and spatial distribution [34]. Many of them develop a shallow root system in the aerated zone of the peat while some produce pneumatophores perpendicular to the taproot to enhance gas exchange [24, 35]. *M. flexuosa* has an extensive root system [29] and like other palm species, it develops an adventitious or fibrous root system growing from the basal nodes of its stem [30]. This system is dense on the surface and in the upper 30 cm of soil, close to the base of the stem [31, 32]. It also branches into an extensive horizontal network, spreading outward for several meters and forms pneumatophores, i.e., aerial roots growing from above the soil surface that facilitate the exchange of gases [29, 33]. In *M. flexuosa* swamp forests, the high pollen abundance of the *Mauritia* taxa (84%) at the top of the peat stratigraphic sequence [36] suggests an important contribution of this species to peat accumulation over time. This high contribution is likely related to the fast productivity of *M. flexuosa* [37] and its dominance over other species over the last c. 400 years [36]. Factors such as the decomposition rate of plant litter (leaf, wood and fine roots) of *M. flexuosa* versus that of trees must also have played a decisive role in peat composition [27] though they have yet to be investigated in these forests. The few studies conducted in tropical peatlands which evaluated the impact of forest degradation on roots demonstrated a decrease in fine root productivity with disturbance [21, 38]. In palm swamp peatlands of the Peruvian Amazon, *M. flexuosa* felling significantly reduced the density of pneumatophores [17, 39]. Fine roots are also expected be affected to a great extent by this degradation which could hereby influence peat formation.

To improve understanding and characterization of C fluxes and budgets in tropical peatlands, we examined fine roots C stocks and dynamics and their environmental controls in a *M. flexuosa* swamp peatland of the Peruvian Amazon according to degradation status. Our experimental design included one undegraded site and two sites with two levels of degradation where fine roots were monitored monthly over 11 months. We made a distinction between trees and *M. flexuosa* palms, and differentiated the later by age class (seedling, juvenile, adult). Our research aimed at addressing the following questions: (1) How does forest degradation affect fine roots C stocks (biomass and necromass for live and dead roots, respectively) and dynamics (productivity, mortality and decomposition rates)? and (2) How do site-scale fine roots productivity and mortality respond to monthly fluctuations in precipitation, water table level and soil temperature?

## Methods

### Site description

The study took place southwest of the city of Iquitos, in the province of Loreto in the Peruvian Amazon. The climate of the region is warm and moist with an average annual temperature and precipitation of 27 °C and 3,087 mm, respectively [40]. Most months exhibit precipitation rates in the range 100 − 300 mm; monthly precipitations > 300 mm are frequent in November, March and April, and less frequent between June and September [40].

The research was conducted in an area of *M. flexuosa* palm swamp forest on peat located nearby the Itaya river, one of the tributaries of the Amazon river [17]. The forest exhibits peat deposits up to 5 m deep, with the 390−400 cm layer radiocarbon dated 2335 years before present [2]. Vegetation development over time has been highly influenced by the flooding regime. Currently, the palm swamp is quasi permanently waterlogged with a water table that rarely falls below 25 cm under soil surface [19] and occasionally floods such as in 1998 (30 cm), 2012 (100 cm) and 2015 (80–150 cm) [19, 36, 41]. The peat in the forest classifies as minerotrophic, i.e., nutrient-rich; and is fed by rainfall and surface or underground river water [17, 42, 43].

**Fig. 1:**
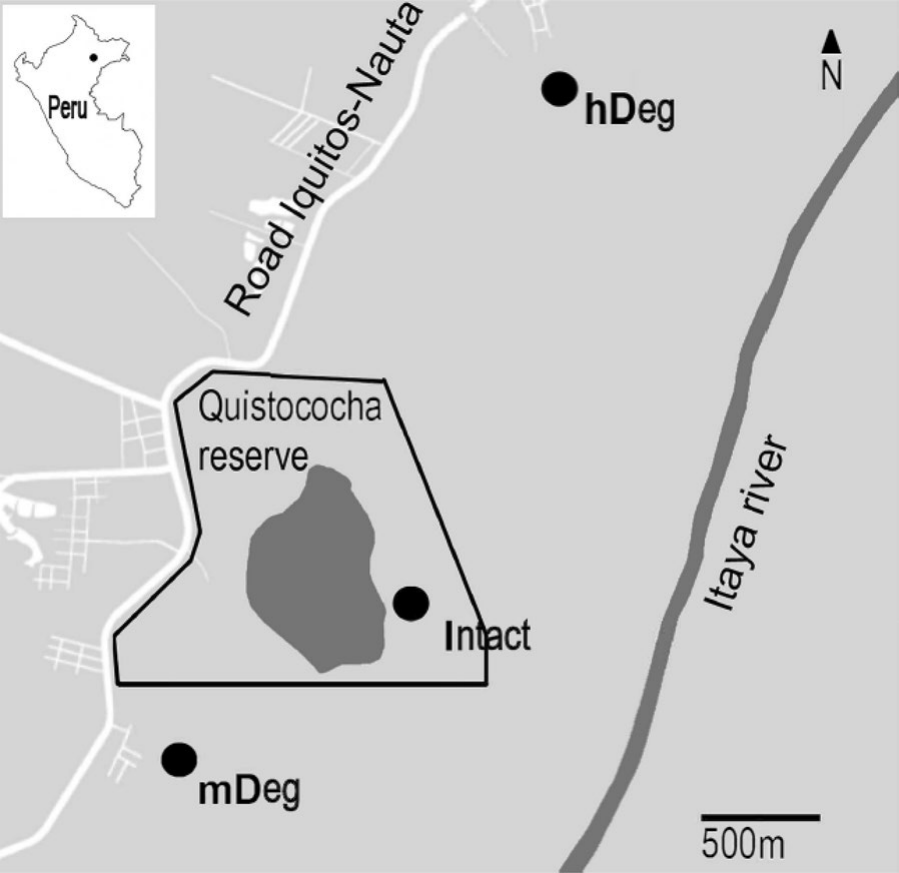
Location of the study sites in the Peruvian Amazon, near the city of Iquitos (black dot on overview map of Peru). The undegraded (Intact) moderately degraded (mDeg) and heavily degraded (hDeg) sites are indicated with black circles. Source: van Lent et al. [17]

**Fig. 2:**
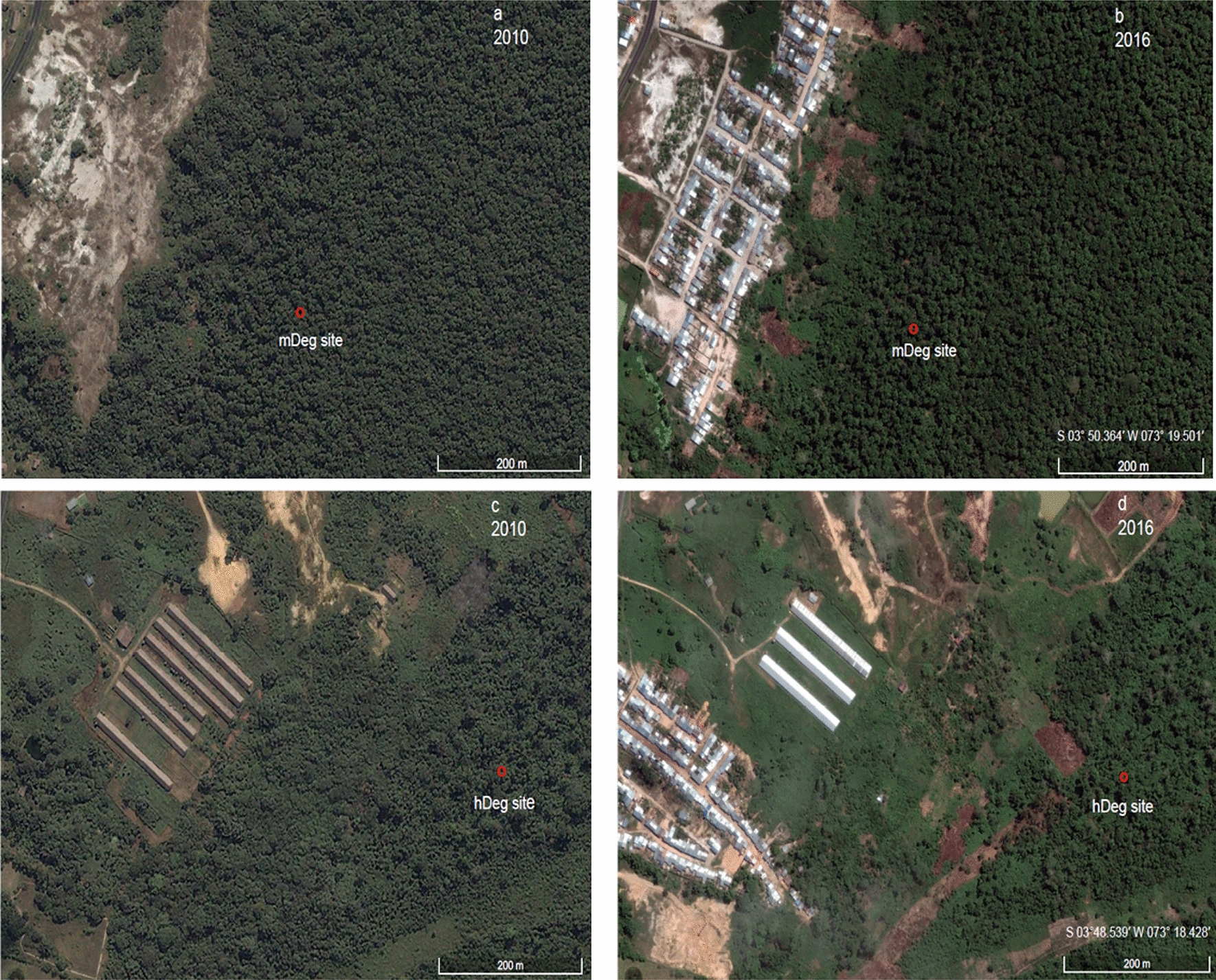
Expansion between 2010 and 2016 of the villages of Las Brizas (panels **a**, **b**) and San Julian (panels **c**, **d**) located nearby the moderately degraded (mDeg) and heavily degraded (hDeg) sites, respectively. Source: Google Earth Pro. Images from June 2010 and August 2016

Three sites were selected within this forest area (Fig. 1). One of them was undegraded and thereafter referred to as intact (Intact), the other sites were used for *M. flexuosa* fruit extraction by destructive harvesting with levels of degradation qualified as moderate (mDeg) and heavy (hDeg). The Intact site was inside the Quistococha protected regional reserve (S 03° 49.949′ W 073° 18.851′) where *M. flexuosa* cutting has been prohibited for over 30 years. The mDeg (S 03° 50.364′ W 073° 19.501′) and hDeg (S 03°48.539′ W 073° 18.428′) sites were located nearby the villages of Las Brizas and San Julian, respectively, which expanded rapidly between 2010 and 2016 (Fig. 2). Both villages developed through unplanned territorial occupation, resulting in the degradation of the forest from timber harvesting and *M. flexuosa* felling for fruit collection; the latter being a main source of income for many local families [44]. Palms are felled manually with a machete at 1 m height with the stump and trunk left on-site. In general, only female fructifying palms are felled but males are also occasionally cut for building paths in the forest and for collecting suri larvae of the palm weevil which are a source of protein for villagers.

The Intact site was a dense forest with closed canopy where *M. flexuosa* stumps and logs were absent. The forest at the mDeg and hDeg sites had, respectively, a reduced and a very open canopy, according to qualitative observation by van Lent et al. [17]. Both degraded sites presented remnant stumps and trunks. Over 2017−2018, no felling of *M. flexuosa* occurred at the Intact and hDeg sites while the felling rate at the mDeg site was 14 individuals ha^−1^ yr^−1^ (Additional file 1: Figs. S1, S2). The absence of felling at the hDeg site denotes the lack of fructifying females. The density (individuals with a diameter at breast height (DBH) > 10 cm) of *M. flexuosa* amounted to 170, 164 and 16 ha^−1^ at the Intact, mDeg and hDeg sites and the density of trees was 1496, 700 and 679 ha^−1^ at the same sites [10]. According to the IVI, *M. flexuosa* was the most important species at the Intact and mDeg sites, while the pioneer *Cecropia membranacea* was the most important one at the hDeg site. Peat depth was 1.92, 1.90 and 0.28 m at the Intact, mDeg and hDeg sites, respectively [10]. The shallower peat at the hDeg site reflects the spatial variability in peat depth inside the forest complex rather than a legacy of degradation. Soils were homogeneous among sites and displayed high concentrations of macronutrients, high phosphorus levels, low bulk densities, high NH_4_^+^ content and low NO_3_^−^ content [19].

### Experimental design

The sampling was designed to evaluate the mass and dynamics of fine roots (< 2 mm in diameter) in surface soil (to a depth of 25 cm) considering the level of forest degradation and distinguishing between two functional plant groups, trees and *M. flexuosa* palms. As a standard, we monitored tree individuals with DBH ≥ 10 cm. Trees were not subcategorized into species because none of them was particularly dominant in undegraded conditions [10] and because of the difficulties in separating and identifying the roots of each tree species. *M. flexuosa* palms were monitored regardless of their diameter since palms are monocots that grow in height almost independently of diameter. Nevertheless, *M. flexuosa* palms were monitored according to age classes by differentiating seedlings from juveniles from adults. These three classes were based on palm height as follows: individuals < 1 m in height were classified as seedlings, individuals ≥ 1 m and < 3 m in height were classified as juveniles, and individuals ≥ 3 m in height were classified as adults (adapted from the study by Holm et al. [45]). The distinction between trees and *M. flexuosa* palms allows site-scaling any changes that degradation induces on the relative proportion of the two plant functional groups and to account for potential differences in fine roots between these groups. The separation *of M. flexuosa* in age classes enables site-scaling the relative proportion of these classes as modified by degradation due to felling of adult individuals and recruitment after felling. It also allows to integrate potential differences between ages in plant allocation to fine roots and their dynamics. However, our experiment assumes no influence of the age of *M. flexuosa* palm on the decomposition rate of its fine roots. Therefore, the root decomposition experiment (see section on fine root decomposition) focuses on adult individuals.

Degradation-driven changes in fine root stocks and fluxes were measured from individuals and site-scaled using forest structure and composition (i.e., plant density per functional group). Only live individuals were monitored hereby assuming a minimal contribution of remnant stumps to fine root inputs to the peat.

### Inventory of the vegetation

The vegetation composition at the sites was inventoried in 2016. Three 50 × 50 m plots per site were established in which all tree with DBH ≥ 10 cm and all *M. flexuosa* seedlings, juveniles and adults were counted. Site density of trees and *M. flexuosa* palms per age class was computed from the sum of individuals across the three plots per site (0.75 ha). Site density was used for site-scaling results from individuals. Other information on vegetation structure and composition, such as species, tree diameter classes and aboveground biomass is presented in the paper by Bhomia et al. [10].

### Determination of fine root mass, productivity and mortality rates

We used the sequential soil coring method [e.g., 46] to determine the stock of fine root biomass (living roots) and necromass (dead roots) and to estimate fine root productivity and mortality rates. This method was preferred over non-destructive techniques which are difficult to implement in flooded wetland ecosystems [47]. As mentioned in the experimental design, sequential coring was performed by distinguishing trees from *M. flexuosa* palms, and considering the age of palms. Fine roots of *M. flexuosa* were easily distinguished from those of trees due to their specific morphological characteristics. Three individuals in each of the four categories (trees and seedling, juvenile, and adults of *M. flexuosa*) were randomly chosen, for a total of twelve individuals per site. Fine root biomass and necromass stocks were monitored monthly around the trunk of each selected individual to a 25 cm soil depth during 11 months (from March 2016 to January 2017) using a 5 cm diameter Russian corer (i.e., a corer specially designed for the collection of peat sediments). A 25 cm depth was chosen as per existing protocols in tropical peatlands [21] and because of the vertical root distribution of trees and *M. flexuosa* palms [48, 49]. The sample points for each individual target tree or palm were positioned circularly around it at a distance of 1 m from the trunk, except for *M. flexuosa* seedlings for which a 0.5 m distance was used. The distance from the trunk was selected for representativeness of the sampled individual and to avoid mixing roots from trees and *M. flexuosa* palms. In the laboratory, roots were washed, separated to a diameter < 2 mm and sorted into live or dead by visual inspection of their characteristics and tensile strength. The roots were dried at 60 °C until they reached a constant mass and weighed.

Fine root production and mortality rates were estimated from the sequential coring data applying the widely used compartmental flow model according to the decision matrix from Fairley and Alexander [50], adapted by Jourdan et al. [51] (Additional file 1: Table S3). The decision matrix estimates fine root production between two sampling dates based on changes in fine root biomass and necromass and losses of fine root necromass due to decomposition as follows:

$${\text{P}}_{{{\text{t}} - 1,{\text{t}}}} = \Delta {\text{B}}_{{{\text{t}} - 1,{\text{t}}}} + \Delta {\text{N}}_{{{\text{t}} - 1,{\text{t}}}} + {\text{D}}_{{{\text{t}} - 1,{\text{t}}}}$$

where P_t−1, t_ is the fine root production rate between month _t−1_ and month _t_; ΔB _t−1, t_ and ΔN _t−1, t_ are the differences in fine root biomass and necromass, respectively, between month _t−1_ and month t; D _t−1, t_ is the necromass decayed over the interval [t − 1, t] which was calculated as [51]:

$${\text{D}}_{{{\text{t}} - {1},{\text{ t}}}} = {\text{N}}_{{{\text{t}} - {1}}} [{1} - {\text{exp}}^{{\left( { - {\text{kt}}} \right)}} ]$$

where N_t−1_ is the necromass at time t − 1 and k is the decay constant generated from the fine root decomposition experiment (see section on fine root decomposition).

Stocks of fine root biomass and necromass as well as rates of fine root productivity and mortality were expressed in C unit using site-specific C contents determined for the corresponding vegetation category (trees, *M. flexuosa* seedlings, juveniles, adults) (see methods in the section on C and nitrogen (N) determinations). Fine root biomass and necromass were averaged across the 11 monitoring months per vegetation category and site. Plot-scale results were computed using the relative densities derived from the vegetation inventory (results presented in Table 1).

**Table 1:** Density of seedlings, juveniles and adults of *M. flexuosa* and of trees at the study sites

Site	Vegetation category	Absolute density (#ha^−1^)	Relative density (%)
Intact	*M. flexuosa* seedlings	28	5
	*M. flexuosa* juveniles	11	2
	*M. flexuosa* adults	182	31
	Trees	362	62
	Sum	582	100
mDeg	*M. flexuosa* seedlings	169	24
	*M. flexuosa* juveniles	35	5
	*M. flexuosa* adults	176	25
	Trees	328	46
	Sum	709	100
hDeg	*M. flexuosa* seedlings	12	7
	*M. flexuosa* juveniles	6	3
	*M. flexuosa* adults	34	19
	Trees	129	71
	Sum	182	100

Seedlings, juveniles and adults were palms < 1 m, 1−3 m and > 3 m in height, respectively. Trees were individuals > 10 cm DBH. Moderately degraded and heavily degraded are abbreviated as mDeg and hDeg, respectively

### Fine root decomposition experiment

The experiment was performed by distinguishing trees from *M. flexuosa* palms regardless of the age of the palm, as mentioned in the general description of the experimental design. The decomposition rate was determined using root decomposition litter bags (20 × 20 cm) constructed from 1.5 mm fiberglass screen. Fine roots were collected from randomly selected adult individuals of *M. flexuosa* and trees using a 5 cm diameter Russian corer. The sampling depth and position from the trunk were the same as for fine root stock measurements and samples were carefully inspected to not mix roots from *M. flexuosa* and trees. At the beginning of the experiment 60 bags were placed at each site, 30 bags containing roots of *M. flexuosa* and 30 bags containing roots of trees. Each bag was filled with 2 g of washed, air-dried fine roots and tagged with an aluminum plate for later identification. The bags were randomly buried at a distance of 1 m from the trunk of a palm or a tree, depending on their content, 15 cm under the soil surface, for a total period of 10 months (March 2016−January 2017). Six bags were monthly collected per site, three with *M. flexuosa* roots and three with dicot tree roots, following the method by Hoyos-Santillan et al. [27]. Upon collection, each bag was gently washed in water, dried at 60 °C until reaching a constant mass, and weighed. The rate of decomposition was determined by regression analysis of the remaining dry mass against time:

$$Y_{t} /Y_{0} = \exp^{{( - {\text{kt}})}}$$

where Y_t_ / Y_0_ is the percentage ratio of remaining dry mass at time t (days) to initial dry mass Y_0_ and k is the decay rate constant (day^−1^). The initial dry mass (1.8 g) was computed from the air-dried mass that was placed in bags (2 g) by applying an oven-dry mass to air-dried mass ratio measured from ten samples (0.9).

### C and N analysis of fine roots

Additional fine root cores for C and N analyses were collected from the soil top 25 cm using a 5 cm diameter Russian corer. Samples were taken at each site around three randomly selected individuals per vegetation category (trees and seedling, juvenile, and adults of *M. flexuosa*). No distinction was made between live and dead roots. The samples were dried at 60 °C to constant mass, milled and homogenized. Their C and N content was determined using the induction furnace method (Costech EA C-N 146 Analyzer).

### Environmental parameters

Precipitation, water table level (WT) and soil temperature were monthly monitored concurrently with the measurements of fine roots stocks. Monthly precipitation was obtained from the daily data of the Puerto Almendra weather station, located near the study sites (National Meteorology and Hydrology Service of Peru—SENAMHI). The WT relative to the soil surface was measured in perforated PVC tubes (5 cm in diameter, 1.5 m in length) installed permanently in the soil. Water depth during flooding was measured with a graduated pole. Soil temperature at 10 cm depth was measured using a portable digital thermometer. Sampling considered the hummock and hollow microtopography of the soil, with nine replicates per microtopographical position for a total of 18 replicates at the Intact site and 18 replicates per microtopographical position for a total of 36 replicates at each of the degraded sites. These environmental parameters were collected as part of a long-term experiment monitoring soil greenhouse gas emissions at the studied sites [19]. We used WT and soil temperature values averaged across hummocks and hollows as fine roots were monitored at a position intermediary between both microtopographies. The results on environmental parameters are briefly presented in Additional file 1: Fig. S4 and are available in more detail in the paper by Hergoualc’h et al. [19]. Here our goal was to investigate how these variables affect root dynamics.

### Statistical analysis

Statistical analysis was performed using the software InfoStat [52], with a probability level of 0.05 to test the significance of effects. Residuals of data were not normally distributed according to the Shapiro-Wilks test; therefore, we used non-parametric tests for data comparison. We applied the Wilcoxon test for comparing the remaining mass of fine roots of *M. flexuosa* versus trees at the end of the decomposition experiment and the Kruskal–Wallis test for comparison of fine root biomass and necromass stocks, fine root productivity and mortality rates and C and N values between sites within a vegetation category and between vegetation categories within a site. Site-scale results were considered significantly different between sites when their value/mean ± standard error did not overlap.

Uncertainty estimates are reported as standard errors. In all calculations, the Gaussian error propagation method was used for propagating uncertainties. This method is adequate for step-by-step calculations that are intended to compute ecological quantities that can be expressed as an analytical equation using addition, subtraction, multiplication and division, such as C stocks or fluxes [53]. The method assumes that uncertainties can be considered to be independent and normally distributed [54]. For addition and subtraction, uncertainties are propagated by quadrature of absolute errors, for multiplication and division propagation by quadrature of relative error [54]. Relationships between monthly fine root productivity or mortality rates and rainfall, WT and soil temperature were tested using site-scale values through linear and non-linear models. We also tested relationships between fine root decay rates and root chemistry (C and N contents, C:N ratio).

## Results

### Vegetation structure and composition along the degradation gradient

The density of *M. flexuosa* adults and trees was slightly lower at the mDeg site than at the Intact site while the density of *M. flexuosa* seedlings and juveniles was, respectively, six and three times higher at the mDeg site than at the Intact site (Table 1). The reduction in vegetation density with increasing degradation was drastic with twice fewer *M. flexuosa* seedlings and juveniles, five times fewer *M. flexuosa* adults and three times fewer trees at the hDeg site compared to the Intact site. Trees dominated the stands at the Intact and hDeg sites while at the mDeg site the share tree: *M. flexuosa* was close to 50:50. The relative densities presented in Table 1 were used for site-scaling fine root biomass, necromass, productivity and mortality rates.

### Stocks of fine root biomass and necromass and rates of fine root productivity and mortality

Fine root C biomass was about twice fine root C necromass for *M. flexuosa* in all age classes (*P* < 0.01), by contrast no such a difference was observed for trees (Table 2). Fine root C biomass in *M. flexuosa* adults was higher at the mDeg site than at the other sites (*P* = 0.0115), while in trees fine root biomass was the lowest at the hDeg site (*P* = 0.0176). Fine root C necromass in trees differed among sites with a higher stock at the mDeg site than elsewhere (*P* = 0.003). Regarding dissimilarities between vegetation categories, fine root C biomass was higher in *M. flexuosa* adults than in seedlings and trees at both degraded sites (*P* = 0.0001 and 0.0002 at the mDeg and hDeg sites); a result which remained the same when merging all sites (*P* = 0.001). Finally, at the mDeg site, fine root C necromass was higher in trees than in *M. flexuosa* adults and seedlings (*P* = 0.0017), which was also valid when the sites were considered all together (*P* = 0.005).

**Table 2:** Fine root biomass and necromass in individuals of *M. flexuosa* and of trees

Site	Vegetation category	Fine root biomass (Mg C ha^−1^)	Fine root necromass (Mg C ha^−1^)
Intact	*M. flexuosa* seedlings	0.48 ± 0.08^A^	0.27 ± 0.05^B^
	*M. flexuosa* juveniles	0.59 ± 0.09^A^	0.37 ± 0.07^B^
	*M. flexuosa* adults	0.58 ± 0.06^Aa^	0.23 ± 0.04^B^
	Trees	0.42 ± 0.07^b^	0.39 ± 0.1^ab^
	Site-scale	**0.48 ± 0.05**^b^	**0.33 ± 0.06**^ab^
mDeg	*M. flexuosa* seedlings	0.42 ± 0.05^Aα^	0.18 ± 0.02^Bα^
	*M. flexuosa* juveniles	0.55 ± 0.07^Aα^	0.29 ± 0.05^Bα^
	*M. flexuosa* adults	1.01 ± 0.12^Abβ^	0.31 ± 0.05^Bα^
	Trees	0.43 ± 0.07^bα^	0.43 ± 0.05^bβ^
	Site-scale	**0.58 ± 0.05**^c^	**0.34 ± 0.03**^b^
hDeg	*M. flexuosa* seedlings	0.34 ± 0.06^Aα^	0.18 ± 0.04^B^
	*M. flexuosa* juveniles	0.49 ± 0.05^Aβ^	0.26 ± 0.07^B^
	*M. flexuosa* adults	0.65 ± 0.10^Aaβ^	0.19 ± 0.03^B^
	Trees	0.23 ± 0.04^aα^	0.26 ± 0.06^a^
	Site-scale	**0.32 ± 0.03**^a^	**0.24 ± 0.04**^a^

Values refer to a soil depth of 0–25 cm

Data are presented as mean ± standard error (n = 33). Mean followed by letters A, B are significantly different between biomass and necromass within a category. Means followed by the letters a and b are significantly different between sites within a category. Means followed by letters α, β, γ are significantly different between categories within a site. No letters are displayed in the absence of a significative difference. Seedlings, juveniles and adults were palms < 1 m, 1 − 3 m and > 3 m in height, respectively. Trees were individuals > 10 cm DBH. Moderately degraded and heavily degraded are abbreviated as mDeg and hDeg, respectively

Site-scale fine root C biomass followed the order mDeg > Intact > hDeg, while site-scale fine root C necromass was higher at the mDeg site than at the hDeg, with the Intact site displaying an intermediate stock not significantly different from the stocks at the degraded sites (Table 2).

**Fig. 3:**
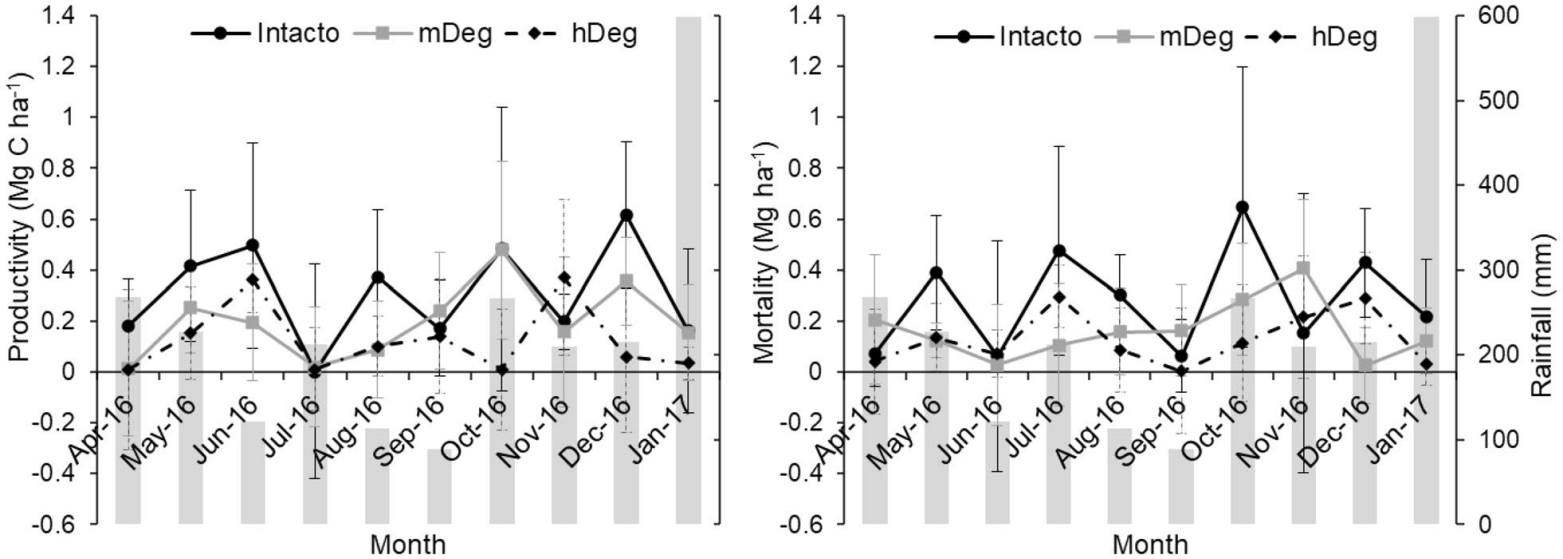
Monthly site-scale fine root productivity (left) and mortality rates (right) and monthly rainfall (grey bars) at the undegraded (Intact), moderately (mDeg) and heavily (hDeg) degraded sites. Error bars are standard errors

**Fig. 4:**
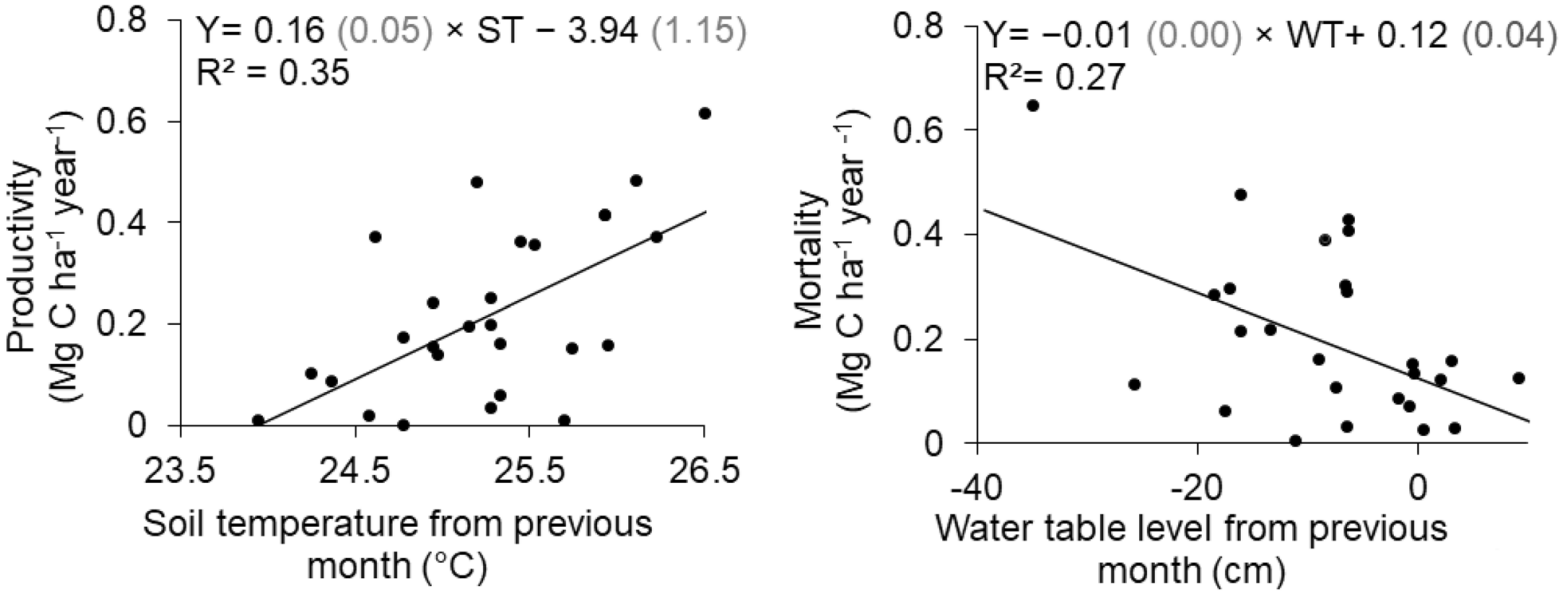
Relationship between site-scale monthly fine root productivity rate and soil temperature (ST) from the previous month (left panel) and between site-scale monthly fine root mortality rate and water table level (WT) from the previous month (right panel) at all sites (n = 30). Values in grey in parenthesis are the standard error of the coefficients of the models. All coefficients of regression are significant at *P* < 0.007. WT and ST data were collected monthly from March 2016 to January 2017 concurrently with the monthly sampling of fine roots. These data were published by Hergoualc’h et al. [19]

Temporal dynamics of fine root productivity and mortality rates are presented at the site-scale (Fig. 3) together with monthly precipitation. Fine root productivity and mortality rates varied unevenly in time across sites. While no trend of maxima was observed, all sites had a fine root productivity rate decreasing to minimum values in July 2016, and a fine root mortality rate decreasing to minimum values in June 2016. Productivity and mortality rates did not exhibit any temporal pattern linked to monthly rainfall variation nor with rainfall of the previous month (*P* > 0.317 in all cases). On the other hand, fine root productivity rate tended to increase linearly with raising soil temperature of the preceding month (*R*^2^ = 0.35, *P* = 0.001), while fine root mortality tended to decrease linearly with increased water table level of the previous month (*R*^2^ = 0.27, *P* = 0.007) (Fig. 4).

Annual productivity and mortality rates of fine roots were similar among sites within a vegetation category or among vegetation categories within a site (Table 3). Site-scale annual productivity rate at the hDeg site was less than half the rate at the Intact site, while the mDeg site displayed an intermediary value. Site-scale annual mortality rates were similar to site-scale annual productivity rates and not significantly different between sites due to their large uncertainties.

**Table 3:** Fine root annual productivity and mortality rates in individuals of *M. flexuosa* and of trees

Site	Vegetation category	Fine root productivity (Mg C ha^−1^ yr^−1^)	Fine root mortality (Mg C ha^−1^ yr^−1^)
Intact	*M. flexuosa* seedlings	2.68 ± 1.6	2.94 ± 1.23
	*M. flexuosa* juveniles	3.57 ± 1.85	2.68 ± 1.71
	*M. flexuosa* adults	3.06 ± 1.13	2.08 ± 0.98
	Trees	4.13 ± 1.91	4.08 ± 2.04
	Site-scale	**3.72 ± 1.24**^b^	**3.37 ± 1.31**
mDeg	*M. flexuosa* seedlings	1.49 ± 0.99	1.60 ± 0.84
	*M. flexuosa* juveniles	2.99 ± 1.32	1.88 ± 1.06
	*M. flexuosa* adults	2.46 ± 2.25	1.70 ± 2.12
	Trees	2.63 ± 1.42	2.26 ± 1.08
	Site-scale	**2.34 ± 0.91**^ab^	**1.95 ± 0.76**
hDeg	*M. flexuosa* seedlings	1.98 ± 1.13	1.61 ± 0.83
	*M. flexuosa* juveniles	1.85 ± 1.24	1.99 ± 1.17
	*M. flexuosa* adults	1.30 ± 2.03	1.73 ± 1.8
	Trees	1.49 ± 1.04	1.46 ± 0.82
	Site-scale	**1.50 ± 0.83**^a^	**1.54 ± 0.68**

Values refer to a soil depth of 0–25 cm

Data are presented as mean ± standard error (n = 10). Mean followed by letters a and b are significantly different between sites. No letters are displayed in the absence of a significative difference. Site-scale values were computed using relative densities from Table 1. Seedlings, juveniles and adults were palms < 1 m, 1−3 m and > 3 m in height, respectively. Trees were individuals > 10 cm DBH. Moderately degraded and heavily degraded are abbreviated as mDeg and hDeg, respectively

### Fine root decomposition

**Fig. 5:**
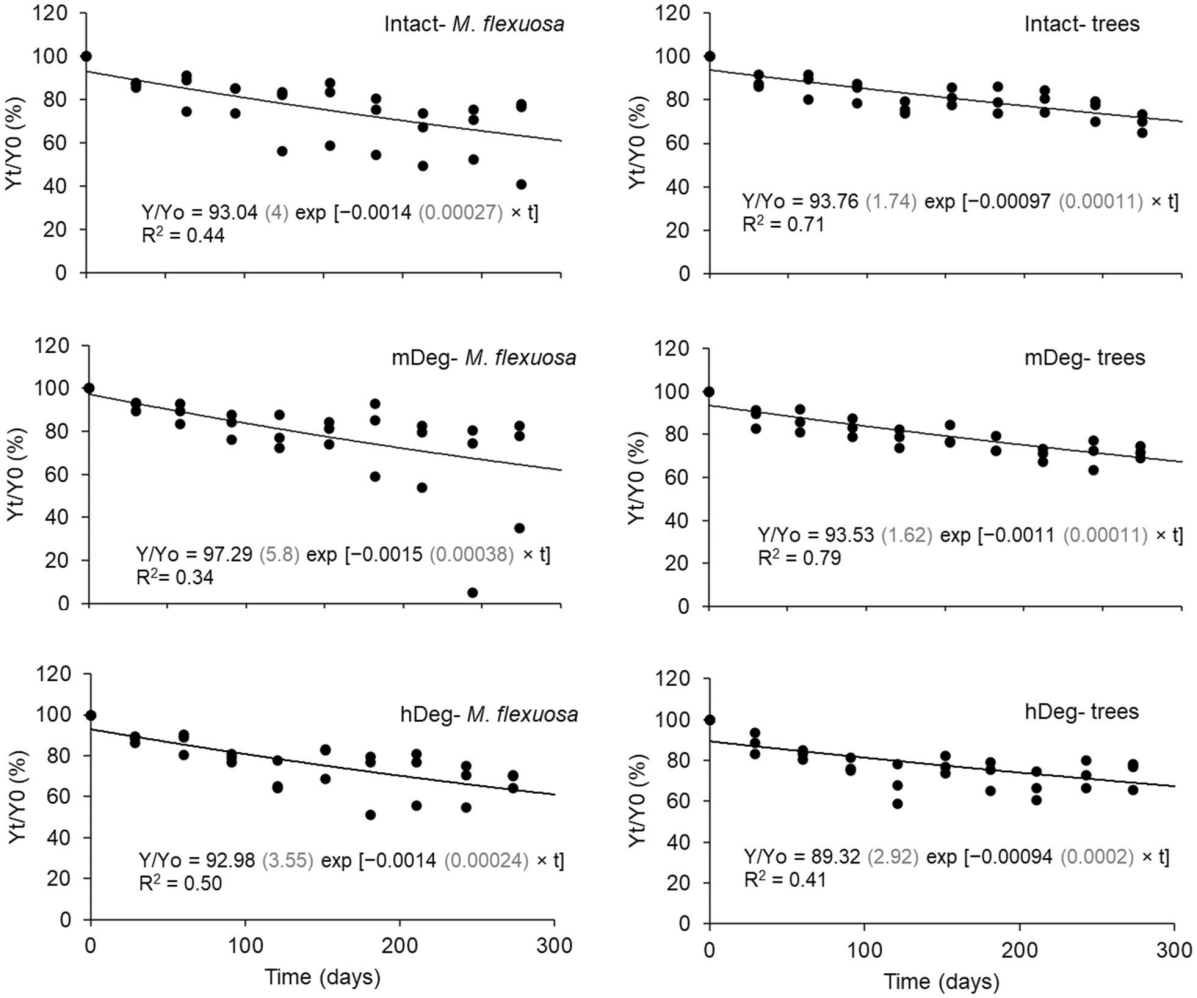
Remaining percentage of initial fine root mass of *M. flexuosa* adults (left) and trees (right) at the undegraded (Intact), moderately (mDeg) and heavily (hDeg) degraded sites. Y_t_/Y_0_ is the percentage ratio of remaining dry mass at time t (days) to initial dry mass Y_0_ and k is the decay rate constant (day^−1^). Values in grey in parenthesis are the standard error of the coefficients of the models. All coefficients are significant at *P* < 0.0004

The remaining percentage of initial fine root mass (2 g) after 300 days (43 weeks) varied between 63.5 ± 13.6% and 74.4 ± 8.1% (Fig. 5). The average remaining percentage across sites and vegetation categories (trees and *M. flexuosa* adults) amounted to 69.2 ± 3.1%. Remaining fine root percentage at day 300 was not different either between sites (*P* = 0.9 and 0.8 for *M. flexuosa* adults and trees, respectively) or between vegetation categories within a site (*P* = 0.8, 0.6 and > 0.99 at the Intact, mDeg and hDeg site, respectively). Decay rates were on average 0.0014 ± 0.0003 d^−1^ and 0.0010 ± 0.0001 d^−1^ for fine roots of *M. flexuosa* adults and trees, respectively. Decay rates were not related to root chemistry (C and N contents, C:N ratio) (*P* > 0.05).

### Root chemistry along the degradation gradient

Fine root C content was higher at the mDeg site than at the hDeg site in all vegetation categories (*P* < 0.025) (Table 4). Fine root N content and C:N ratio were, respectively, higher and lower at the hDeg site than at the Intact site in *M. flexuosa* seedlings (*P* = 0.0071 and 0.0089, respectively). At the hDeg site, fine root N content was lower in *M. flexuosa* seedlings and juveniles than in trees (*P* = 0.0324). At the same site, the C:N ratio of fine roots was higher in *M. flexuosa* juveniles than in trees (*P* = 0.0415).

**Table 4:** Fine root C content, N content, and C:N ratio

Site	Vegetation category	C (%)	N (%)	C:N
Intact	*M. flexuosa* seedlings	49.11 ± 0.32^ab^	0.75 ± 0.06^a^	65.99 ± 5.44^b^
	*M. flexuosa* juveniles	48.74 ± 0.33^ab^	0.77 ± 0.06	64.19 ± 4.9
	*M. flexuosa* adults	48.35 ± 0.52^ab^	0.75 ± 0.05	64.89 ± 4.92
	Trees	49.14 ± 0.37^ab^	0.80 ± 0.03	62.01 ± 2.32
mDeg	*M. flexuosa* seedlings	50.10 ± 0.45^b^	0.97 ± 0.05^ab^	51.77 ± 2.2^ab^
	*M. flexuosa* juveniles	49.66 ± 0.19^b^	0.91 ± 0.06	55.07 ± 3.6
	*M. flexuosa* adults	49.65 ± 0.77^b^	0.84 ± 0.06	59.94 ± 4.71
	Trees	49.99 ± 0.39^b^	0.93 ± 0.12	55.7 ± 7.62
hDeg	*M. flexuosa* seedlings	46.91 ± 0.69^a^	1.05 ± 0.01^bαβ^	44.98 ± 0.66^aαβ^
	*M. flexuosa* juveniles	46.73 ± 0.18^a^	0.88 ± 0.07^α^	54.02 ± 4.59^β^
	*M. flexuosa* adults	46.00 ± 0.22^a^	0.95 ± 0.08^α^	49.04 ± 4.56^αβ^
	Trees	46.93 ± 0.71^a^	1.50 ± 0.16^β^	32.08 ± 3.50^α^

Data are presented as mean ± standard error (n = 3). Means followed by letters a and b are significantly different between sites within a category. Means followed by letters α, β are significantly different between categories within a site. Seedlings, juveniles and adults were palms < 1 m, 1−3 m and > 3 m in height, respectively. Trees were individuals > 10 cm DBH. Moderately degraded and heavily degraded are abbreviated as mDeg and hDeg, respectively

## Discussion

### Effect of forest degradation on vegetation composition and chemical properties

Anthropogenic disturbance altered not only the densities of *M. flexuosa* and trees, but also the age distribution of *M. flexuosa* palms. At the hDeg site, the densities of *M. flexuosa* seedlings, juveniles, and adults and the density of trees decreased by, respectively, 56%, 43%, 81% and 64% as compared to densities at the Intact site (Table 1). This reduction agrees with observations by Horn et al. [13] along *M. flexuosa* stands of varying level of felling. Their results indicate a decrease in *M. flexuosa* seedlings, juveniles, and adults resulting from degradation of 72%, 71%, 78% (estimates computed using densities from their two sites with a *M. flexuosa* male to female ratio of 1 (sites 2, 11) as an undisturbed reference and from the site with a ratio of 10 (site 12) as a highly degraded stand). Similar as at the highly degraded site observed by Horn et al. [13], the rapid and intense felling of *M. flexuosa* female adults at the hDeg site (see history in Fig. 2) induced a drastic reduction of seedlings and juveniles seemingly suppressing its potential for regeneration. Though our experiment did not differentiate males from females, it is likely that the few remaining adults at the hDeg site were males; as suggested the relationship by Horn et al. [13] between low densities of adult females and low densities of seedlings. The strong disturbance at that site as visible in situ by its very reduced canopy cover was also evidenced by its floristic composition with *Cecropia membranacea*, a pioneer species ranking first in IVI and *M. flexuosa* ranking only in the 6th position [10].

At the mDeg site, the density of trees and *M. flexuosa* adults decreased only very slightly as compared to densities at the Intact site (− 9% and − 3%, Table 1). On the other hand, densities of *M. flexuosa* seedlings and juveniles were, respectively, six and three times higher than at the Intact site. These results contrast with observation by Horn et al. [13] who found no trend in seedling density and a decrease in juvenile density with increasing degradation (using the *M. flexuosa* male to female ratio as a degradation metric; Additional file 1: Fig. S5). The high seedling and juvenile densities indicate that moderate degradation as practiced at our site (i.e., with a *M. flexuosa* felling rate of 14 adults ha^−1^ yr^−1^) favors *M. flexuosa* recruitment dynamics, probably as the result of increased light availability in gaps created by logging. The existence of seed and seedling banks waiting for appropriate light conditions for their growth has been widely recognized for many forest species under different conditions [55–57]. *M. flexuosa* establishment strategies rely on a combination of seed recalcitrance and seed dormancy, which maintain seed and seedling banks [58, 59]. This species is also shade-intolerant and its successful regeneration depends on light accessibility, notably in gaps created by natural disturbances [60].

The higher C content in fine roots of *M. flexuosa* and trees at the mDeg site than at the hDeg site (with the Intact site presenting intermediary values) (Table 4), suggests an enhanced C allocation to the root system under moderate degradation. Changes that increase stand level photosynthesis over time are known to increase C allocation to fine roots and other belowground pools [e.g., 61, 62]. At the hDeg site, the higher N content in fine roots of trees as compared to contents for *M. flexuosa* seedlings and juvenile and their lower C:N ratio than that of *M. flexuosa* juveniles (Table 4) suggest the presence of N-fixing species in the pool of tree species.

### Fine root productivity and mortality rates and their temporal variation

Site-scale annual fine root productivity rates (1.5−3.7 Mg C ha^−1^ yr^−1^) are comparable with the average fine root productivity reported by Chimner and Ewel [21] for a tropical forested peatland (2.3 Mg C ha^−1^ yr^−1^) and relatively higher than values measured by Finér and Line [63], Baker et al. [64], and Yuan and Chen [65] (0.6−2.8 Mg C ha^−1^ yr^−1^) in northern peatland forests. Fine root mortality rates at site scale (1.5−3.4 Mg C ha^−1^ yr^−1^) were above the average for peat swamp forests of Southeast Asia computed by Hergoualc'h and Verchot [66] (1.5 Mg C ha^−1^ year^−1^).

To understand temporal variations of fine root dynamics, we tested the control that environmental factors exert on monthly fine root productivity and mortality rates. We found weak but significant relationships indicating that site-scale monthly fine root productivity rate tends to be positively and linearly related with soil temperature of the preceding month and monthly fine root mortality rate tends to be negatively linearly related with the water table level of the preceding month (Fig. 3). A one-month lag between variation in environmental variables and the response of vegetation growth and dynamics has been documented by many authors [67–71]. In particular, these authors suggested that radiation in the previous month enables photosynthesis for C fixation and provides the essential conditions for growth in the following month. The fine root productivity rate−soil temperature relationship concurs with current understanding on temperature stimulation of plant photosynthesis, C allocation to roots, and microbial processes improving nutrient availability for plant productivity [72–75]. On the other hand, the decrease in fine root mortality as the water table rises is less trivial to interpret. There is no indication that this pattern is common in peatland forests, however wetland species are known to have developed structures in their shallow root system that allow them to survive and thrive under flood conditions [24, 34, 76]. So, we speculate that these structures are negatively affected by desiccation when the water table decreases, causing their death. The formation of pneumatophores, for instance, is impeded in flooded forest subjected to regular and high amplitude fluctuations of the water table [34, 77, 78]. Our result suggests that species growing in these palm swamp forests are quite tolerant to high water saturation levels, and that their fine roots could be seriously affected by prolonged decreases in soil moisture due to the lowering of the water table.

### Biomass, necromass and decomposition of fine roots

Fine roots and their temporal dynamics have been poorly investigated in tropical peatlands despite their importance in C and nutrient cycling. Fine root biomass (0.2 − 1.0 Mg C ha^−1^) and necromass (0.2 − 0.4 Mg C ha^−1^) (Table 2) were significant under intact and degraded conditions, as also shown for tropical forested peatlands of Micronesia [21] (fine root biomass of 1 Mg C ha^−1^ in the soil top 30 cm). The fine root biomass to necromass proportion differed according to plant functional group (Table 2). *M. flexuosa* fine roots consistently presented higher biomass than necromass regardless of palm age and degradation level, corroborating the capacity for the root system of this species to thrive in waterlogged conditions [29]. On the contrary, trees, which were floristically different between sites [10], had similar fine root biomass and necromass at all sites. The much larger fine root biomass of *M. flexuosa* than that of trees across sites is in agreement with results by Haggar and Ewel [79] who found that biomass allocation to resource-capturing structures such as fine roots is higher for monocots than for dicot trees, with the later investing more in support structures, particularly coarse roots.

The differences between vegetation categories in fine root C biomass or necromass at the degraded sites and the absence of such differences at the Intact site (Table 2) suggest that degradation affected fine roots dynamics over time. Likewise, the lower fine root biomass of trees at the hDeg site as compared to that at the Intact site (Table 2) indicates a change in fine root dynamics, probably associated with the shift in floristic composition at this site [10]. Site-scale fine root biomass and necromass decreased with high degradation as the result of reduced species-specific root mass combined with reduced vegetation density (Table 1).

Fine root decomposition was slow and uniform among sites and vegetation type. Our 69.2% cross-treatment average of remaining percentage after 300 days is in accordance with the 72% remaining percentage after 252 days found in a tropical forested peatland of Micronesia [21]. Average decay rates of fine roots for *M. flexuosa* adults (*k* = 0.0014 d^−1^) and trees (*k* = 0.0010 d^−1^) (Fig. 4) are of the same magnitude of the average decay rate (*k* = 0.0016 d^−1^) measured in *Raphia taedigera* palm swamp peatlands of Panama [27]. In agreement with findings by Chimner and Ewel [21] and Hoyos-Santillan et al. [27], fine roots decay rates were much lower than the average decay rate of leaf litter (*k* = 0.0027 d^−1^) measured at our sites by van Lent [17]. Therefore, our results reinforce the conclusion of the critical role of fine roots in building peat material over time in tropical environments. Root chemistry, in particular the C:N ratio, is an important factor controlling root decomposition [21, 80]. The similar fine root C:N ratios among plant functional types and sites (Table 4) and the absence of a relationship between C:N ratio and decay rates thus coincides with the absence of differences in decay rates between plant functional types and sites.

## Conclusions

Fine roots are an important component of the C budget of peat soils, accounting for 17–23% of total C inputs to the peat in tropical forests [25, 26]. As demonstrated for peatlands of Southeast Asia, forest degradation through logging and draining induces an average decrease in fine root C inputs by as much as 50% [25]. Felling of *M. flexuosa* palms and trees reduced site-scale fine root productivity by 37% and 42% at the mDeg and hDeg sites, respectively (Table 3). It also decreased site-scale fine root mortality rates by 60% and 54% at the same sites, albeit not significantly due to large errors associated to values. These results combined with findings by van Lent [39] of lower aboveground litter inputs and larger soil organic matter losses through heterotrophic respiration, especially at the heavily degraded site demonstrate that degradation of this ecosystem can negatively and strongly affect peat accumulation.

Future research may consider repeat this type of experiment over a longer time scale based on a larger sample size and evaluate how soil microtopography (hummock, hollow) may affect root C stocks and fluxes. Furthermore, given the local scope of the study additional work is needed to test the response of fine roots to degradation elsewhere in the Peruvian Amazon or in tropical peat swamp forests in other regions where logging activities are taking place. The role that fine roots play in peat C budgets remains largely understudied and deserves much more attention, especially in tropical climates.

## Supplementary Information

**Additional file 1: Figure S1.** Location and length (m) of the transects for monitoring M. flexuosa felling at the moderately degraded site (mDeg). Lateral visibility within each transect was 6 m for AB; 10 m for BC; 7 m for CD, CE and EF; and 5 m for FB. Source: Google Earth Pro. Image from February 2017. **Figure S2.** Monthly rate of M. flexuosa cutting at the moderately degraded (mDeg) site. **Figure S3.** Sequential coring to estimate fine root production and mortality rates. **Table S3.** Decision matrix for estimating fine root production and mortality according to Fairley and Alexander [50], adapted by Jourdan and others [51]. **Figure S4.** Monthly mean water table level and soil temperature at the Intact, moderately (mDeg) and heavily (hDeg) degraded sites. Error bars are standard error. **Figure S5.** Relationship between M. flexuosa male: female ratio and M. flexuosa density in palm swamps stands of the Peruvian Amazon. The figure is based on the data by Horn et al. [13]. Seedling (< 1 m in height), juveniles (1−3 m in height) and adults (> 3 m in height).

## Data Availability

Details of all data and materials used in the analysis are available in the main text or on request of the corresponding author.

## Abbreviations

C: Carbon
DBH: Diameter at breast height
hDeg: Heavily degraded
IVI: Importance value index
mDeg: Moderately degraded
N: Nitrogen
NH4^+^: Ammonium
NO3^−^: Nitrate
PVC: Polyvinyl chloride
WT: Water table level
