# Supplementary material for: Degradation-driven changes in fine root carbon stocks, productivity, mortality, and decomposition rates in a palm swamp peat forest of the Peruvian Amazon
Source: Carbon Balance Manag. 2021 Oct 29;16:33. doi: 10.1186/s13021-021-00197-0 (PMC8555211; doi:10.1186/s13021-021-00197-0)
Supplement: Supplementary file 1 — Additional file 1: Figure S1. Location and length (m) of the transects for monitoring M. flexuosa felling at the moderately degraded site (mDeg). Lateral visibility within each transect was 6 m for AB; 10 m for BC; 7 m for CD, CE and EF; and 5 m for FB. Source: Google Earth Pro. Image from February 2017. Figure S2. Monthly rate of M. flexuosa cutting at the moderately degraded (mDeg) site. Figure S3. Sequential coring to estimate fine root production and mortality rates. Table S3. Decision matrix for estimating fine root production and mortality according to Fairley and Alexander [50], adapted by Jourdan and others [51]. Figure S4. Monthly mean water table level and soil temperature at the Intact, moderately (mDeg) and heavily (hDeg) degraded sites. Error bars are standard error. Figure S5. Relationship between M. flexuosa male: female ratio and M. flexuosa density in palm swamps stands of the Peruvian Amazon. The figure is based on the data by Horn et al. [13]. Seedling (< 1 m in height), juveniles (1−3 m in height) and adults (> 3 m in height). [file 13021_2021_197_MOESM1_ESM.docx]

ADDITIONAL INFORMATION

**Degradation-driven changes in fine root carbon stocks, productivity, mortality, and decomposition rates in a palm swamp peat forest of the Peruvian Amazon**

**List of Authors**: Nelda Dezzeo^1,2^, Julio Grandez-Rios^1,3^, Christopher Martius^4^, Kristell Hergoualc’h*^1^

**Institutional affiliations**:

^1^ Center for International Forestry Research (CIFOR), Lima, Peru

^2^ Venezuelan Institute for Scientific Research (IVIC), Caracas, Venezuela

^3^ Universidad Nacional de la Amazonia Peruana (UNAP), Loreto, Peru

^4^ Center for International Forestry Research (CIFOR), Bonn, Germany

*** Corresponding author:** Kristell Hergoualc’h. Center for International Forestry Research (CIFOR) c/o Centro Internacional de la Papa (CIP), Av. La Molina 1895, La Molina, Apdo Postal 1558, 15024 Lima Peru

e-mail: k.hergoualch@cgiar.org

***S1: Monitoring of M. flexuosa felling rate at the moderately degraded (mDeg) site from September 2017 through July 2018***

The rate of *M. flexuosa* felling was monitored at the mDeg site over 10 months, from September 2017 through July 2018. It was not studied at the other two sites where no *M. flexuosa* felling occurred during the measurement period since the Intact site was located in a protected area, and the high degradation at the hDeg site seemingly only left *M. flexuosa* unfructifying males.

At the mDeg site, we established a transect along which the number of downed *M. flexuosa* trunks was counted monthly. The monthly rate of felling was computed as the difference of downed trunks observation between a month and the previous month. The annual rate of *M. flexuosa* felling was calculated by annualizing the sum of monthly rates over ten months. The monitoring was conducted along six subtransects (AB, BC, CD, CE, EF, and FB) representing a total monitoring area of 1.4 ha (Fig. S1). The length of the subtransects and the visibility distance to each side of the subtransects were measured with a metric tape. These results were used for calculating the area per subtransect: 0.22, 0.18, 0.42, 0.14, 0.33, 0.09 ha for AB, BC, CD, CE, EF and FB, respectively. Two of the subtransects (AB and CD) were paths used by community members for felling *M. flexuosa* palms; the remaining subtransects were located outside of felling paths. The transect comprised 46% of its area within felling paths, 54% of it outside of felling paths. Therefore, the felling rate monitored over this transect can be considered as representative of felling rates inside the whole forest site according to common practices by the surrounding village members.


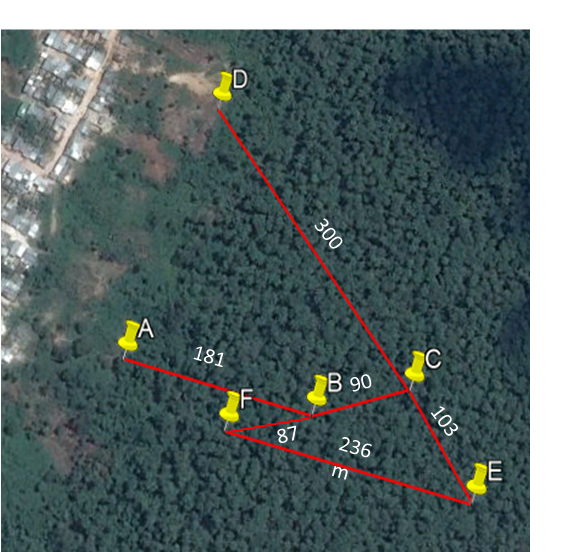


Figure S1. Location and length (m) of the transects for monitoring *M. flexuosa* felling at the moderately degraded site (mDeg). Lateral visibility within each transect was 6 m for AB; 10 m for BC; 7 m for CD, CE and EF; and 5 m for FB. Source: Google Earth Pro. Image from February 2017.

The rate of *M. flexuosa* felling amounted to 14 individuals ha^−1^ y^−1^ in the period from September 2017 until July 2018. *M. flexuosa* palms were felled mainly in the months of April and June (Figure S2).


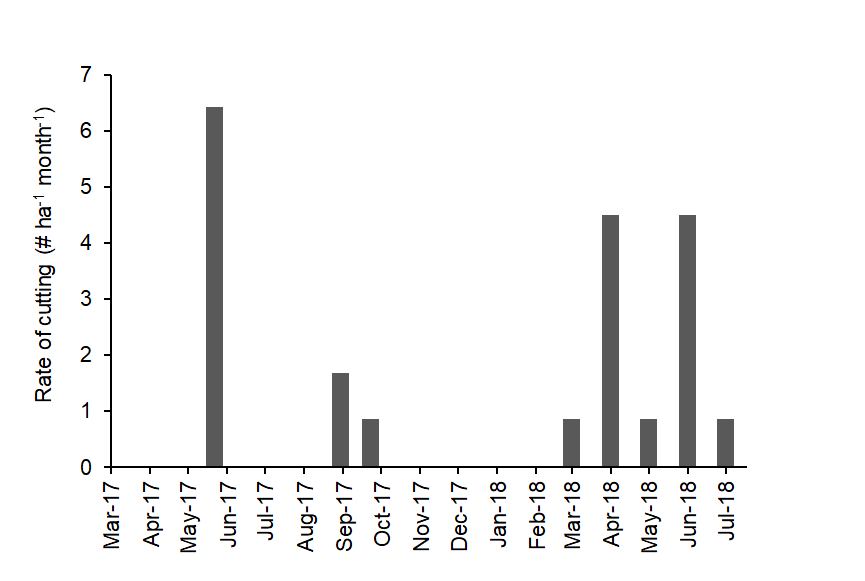


Figure S2: Monthly rate of *M. flexuosa* cutting at the moderately degraded (mDeg) site.

***S3: Sequential coring to estimate fine root production and mortality rates.***

Table S3. Decision matrix for estimating fine root production and mortality according to Fairley and Alexander [50], adapted by Jourdan and others [51].

|  | Biomass increase | Biomass decrease | |
| --- | --- | --- | --- |
|  |  | ΔB < ΔN | ΔB > ΔN |
| Necromass increase | P = ΔB + ΔN + D  M = ΔN | P = ΔB + ΔN + D  M = ΔN | P = 0  M = − ΔB |
| Necromass decrease | P = ΔB  M = 0 | P = 0  M = − ΔB | P = 0  M = − ΔB |

B is the fine root biomass; N is the fine root necromass; P and M are the fine root production and mortality rate, respectively, between two sampling dates; D is the necromass decomposed between two sampling dates; ΔB and ΔN are the fine root biomass and necromass change, respectively, between two sampling dates.

***S4: Fluctuations of water table level and soil temperatures over the study period***

Water table levels tend to be higher at the mDeg site (−18.4 - 9.3 cm) than at the hDeg (−28.0 - 1.9 cm) and at the Intact (−33.0 - −4.0 cm) sites. Soil temperature was variable over the study period, with monthly means fluctuating between 25.2 - 27.7°C at the Intact site, 25.3 - 26.8°C at the mDeg site and 24.9 - 28.1°C at the hDeg site (Figure S4).


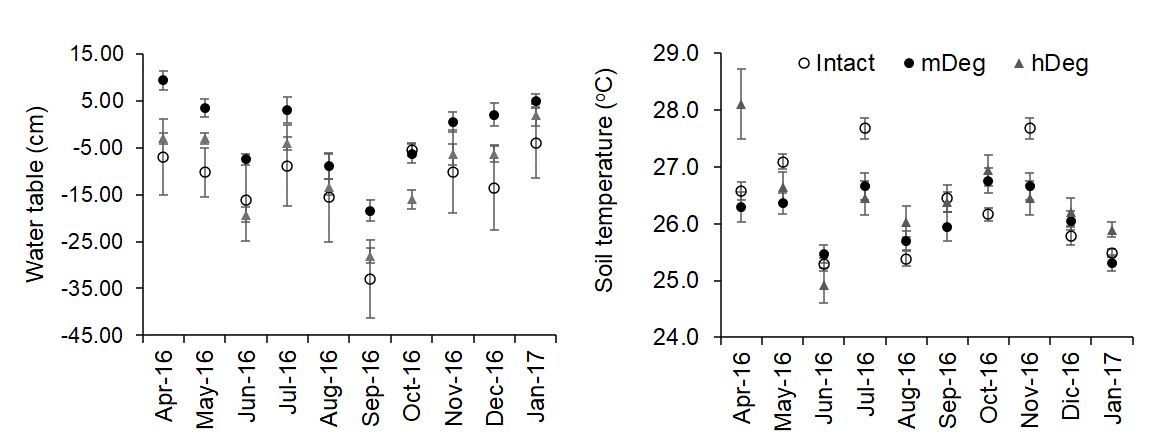


Figure S4. Monthly mean water table level and soil temperature at the Intact, moderately (mDeg) and heavily (hDeg) degraded sites. Error bars are standard error.


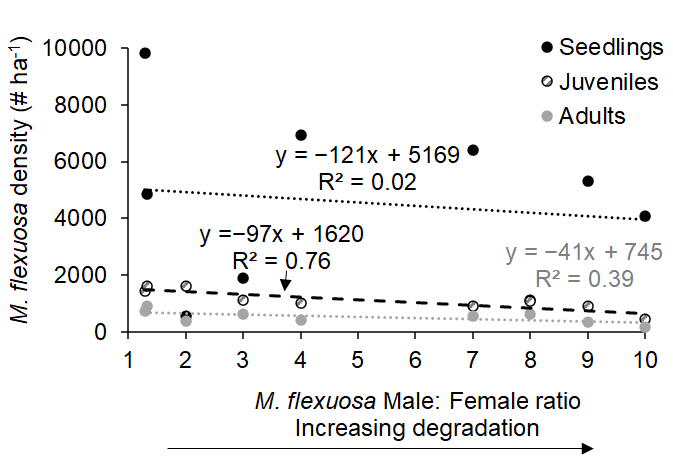


Figure S5. Relationship between *M. flexuosa* male: female ratio and *M. flexuosa* density in palm swamps stands of the Peruvian Amazon. The figure is based on the data by Horn et al. [13]. Seedling (< 1 m in height), juveniles (1−3 m in height) and adults (> 3 m in height).
